# Linking undergraduates’ future work self and employability: a moderated mediation model

**DOI:** 10.1186/s40359-024-01530-1

**Published:** 2024-03-18

**Authors:** Yaju Ma, Lingyan Hou, Wenjing Cai, Xiaopei Gao, Lin Jiang

**Affiliations:** 1 School of Education, Weinan Normal University, Weinan, China; 2 School of Public Affairs, University of Science and Technology of China, Hefei, China; 3 Department of Management & Organisation, Vrije Universiteit Amsterdam, Amsterdam, Netherlands

**Keywords:** Future work self, Employability, Career exploration, Job market knowledge

## Abstract

**Background:**

The career intentions of students play a crucial role in shaping the growth of the hospitality and tourism industry. Previous research underlines the significance of future work self in predicting outcomes related to one’s career. However, there is limited knowledge regarding the precise ways, timing, and conditions under which the future work self of undergraduate students can enhance their employability.

**Methods:**

This paper aims to address the existing research gap by employing career construction theory and self-determination theory to propose a moderated mediation model—i.e., career exploration serves as a mediator and job market knowledge functions as a moderator in the relationship between future work self and employability. We conducted two independent studies (i.e., an experimental study and a time-lagged field study) to test the proposed model. Specifically, in Study 1 we employed an experimental research design to recruit 61 students majoring in tourism management to participate. They were randomly assigned to two scenarios (future work self: high vs. low), and we manipulated different levels of future work self by means of scenario descriptions. In Study 2, we used the time-lagged research design to collect data via submitting questionnaires among 253 Chinese undergraduates who majored in hospitality and tourism at a university in the middle area of China.

**Results:**

The results indicate a positive correlation between undergraduates’ future work self and their employability. Furthermore, this relationship is mediated by a mediator of career exploration. It is important to note that this mediating relationship is also contingent upon the moderator variable of undergraduates’ job market knowledge when considering the impact of career exploration on employability.

**Conclusion:**

The findings contribute to enriching the current understanding of the positive effects of future work self on undergraduates’ desirable outcomes in employability.

**Supplementary Information:**

The online version contains supplementary material available at 10.1186/s40359-024-01530-1.

## Introduction

Human resources, as a significant determinant of the growth of the hospitality industry, highlights the importance of undergraduates specializing in the hospitality and tourism industry [1]. A recent review correspondingly indicated that students’ career intentions determine the need for hospitality and tourism management programs from the perspective of the hospitality and tourism industry [2]. In light of the uncertainties surrounding employment opportunities and the substantial decrease in job openings during the transition from school to work due to the COVID-19 pandemic [3], universities are prioritizing the development of undergraduates’ employability to help them gain employment and be successful in their chosen occupations after graduation [4], especially for educating undergraduates’ majored in hospitality and tourism relevant areas. Given the heightened risk of COVID-19 transmission within the hospitality sector [5], these undergraduates’ pessimistic perception about the current and future workforce of the tourism industry significantly influences their career attitudes and behaviors toward their future jobs [6, 7]. In addition, some research findings have suggested that newcomers, especially graduates, encounter higher occurrences of job mismatching and underemployment [8]. Consequently, with the goal of increasing undergraduates’ capabilities to transition from school to work, scholars have empirically demonstrated that developing students’ personal characteristics (e.g., proactivity, career adaptability, and knowledge, skills, and attitudes) increases their employability in the future job market [9].

Scholars argue the necessity for universities to not only focus on improving students’ employability skills but also on fostering their career motivation [10]. For students who have not yet entered the workplace, their future work selves—i.e., their thoughts and hopes about their future jobs—are the driving force behind their proactive career preparations and early job search behaviors [11]. Future work self is an important motivational resource for proactive career behavior, individuals with a high level of future work self tend to engage in proactive career behaviors (e.g., career planning) toward a better employment status in the future [12]. Existing research suggests that motivation is a significant and substantial predictor of student employability [13, 14]. However, empirical research on the relationship between students’ future work self, an important motivational resource, and perceived employability is still lacking, and the mechanism of whether and how future work self act on students’ perceived employability remain unclear. Thus, this study aims to address the following research question: *whether, how and when undergraduates’ future work self contributes to their perceived employability*.

**Fig. 1:**
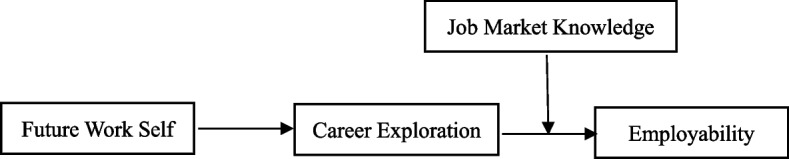
Conceptual model

Career construction theory proposes that the concept of future work self serves as a source of motivation, encouraging individuals to invest more effort in career-related behaviors by developing goals and strategies for their future work [11, 15, 16]. Meanwhile, self-determination theory emphasizes that intrinsic motivation (i.e., future work self) is positively associated with key attitudes and behaviors [17]. Specifically, a strong future work self, which represents a significant intrinsic motivation, enables individuals to adopt an exploratory approach in navigating the uncertainty surrounding their future work. This is achieved by developing and accomplishing self-derived goals and strategies that contribute to positive work and career outcomes [18]. Therefore, we expect that undergraduates with a salient future work self would have a self-starting motive to explore their career toward boosting their success in the future job market (i.e., employability). Furthermore, as career construction theory’s suggestion that environmental factors can influence an individual’s career development [19], those who are aware of relevant contextual cues can actively process career-related information and advance their careers [20]. Accordingly, students who possess comprehensive knowledge about the job market are more likely to be concerned about their future career trajectory and engage in more activities related to career exploration, all in the pursuit of increasing their employability in the future job market, compared to those lacking such knowledge [21]. Taking all of this into consideration, we propose our hypothesized model in Fig. 1.

With this research, we make two main contributions to the literature. First, by linking the positive association between the future work self and undergraduates’ perceived employability, we enrich the current academic understandings of career construction theory [19] and self-determination theory [22]. That is, we empirically link the motivational benefits of the future work self to undergraduates’ employability in the future job market. Meanwhile, with the guidance of two distinct theoretical perspectives [23–25], we propose and test career exploration, a central process in students’ career development [26], as a key mediator in explaining the relationship between the future work self and employability. In this vein, the results enhance the present comprehension of the influence that the future work self has on employability. Second, by examining job market knowledge as a boundary condition for the indirect relationship between future work self and employability in undergraduates’ career exploration, we included the potential moderator of the acquisition of job-related knowledge in the study, which helped to elucidate the role of future work self in relation to career exploration from the perspective of individual dependency characteristics.

## Literature Review and Hypothesis Development

### Future work self and perceived employability

In the age of VUCA, the flexible employment relationship and the blurring of organizational boundaries are making individuals’ careers discontinuous and “boundaryless” [27]. As career paths become more uncertain, individuals need to engage in increasingly proactive career behaviors to enhance their employability [28] and access jobs and careers that match their values and needs [29]. To better prepare for the transition from school to work [30] and to develop employability in a changing organizational environment, it is critical for students to proactively shape their career future and actively manage their careers [31].

With reference to the research of Rothwell, Herbert, & Rothwell (2008), we defined the employability as the ability perceived by university students to maintain existing jobs and obtain desired jobs. It is categorized into internal and external dimensions [32]. Specifically, internal employability refers to the self-evaluation and career value perception felt by employees in the organization, while external employability refers to the willingness and ability of employees to transfer to other organizations, reflecting the value of employees in the external labor market [33]. Scholars have also suggested that the perception of employability is influenced by self-concept [34]. In other words, students’ personal traits play a significant role in predicting their future employability perception [35]. When students become job seekers, they begin to focus more on their future career direction than before and are concerned about their future employability [36]. We consider that students’ perceived employability while constructing their careers is closely related to their future work selves.

The future work self is a conceptual representation of an individual’s aspirations and hopes for their future self in the work domain [24]. Compared to the general concept of the “possible self”, the future work self is future-oriented, work-related, and includes the two attributes of salience and elaboration [37]. Specifically, future work self-salience refers to the degree to which the person’s future work self is clear and imaginable. Future work self-elaboration can be extrapolated from the complex and detailed descriptions of representations of the future self [24]. According to career construction theory, individuals should consider their past memory, current experience, and future aspirations to make their career behavioral choices [19]. The future work self potentially expands undergraduates’ aspirations and develops their thinking about future career possibilities [16], which enables them to proactively prepare for enhancing their employability [38]. According to self-determination theory, the future work self serves as a motivational career resource that can motivate students to engage in current goal-setting and goal-striving behaviors to achieve a desired future [22, 39]. Students who have a higher level of salience and elaboration about their future work self can not only clearly depict the image of their future work [40] but also take the initiative to learn job-related knowledge and skills needed in career development [12] to purposefully enhance a series of comprehensive abilities and strengthen their employability [41]. It follows that the future work self plays a motivating role in increasing students’ perceived employability. Accordingly, we propose the hypothesis as followed:

Hypothesis 1. Future work self is positively related to an undergraduate’s perceived employability.

### The mediating role of career exploration

Career exploration is the most essential stage in the career development of students [42]. Sufficient and proactive exploration contributes to better self-awareness [43] and greater career-related outcomes. Career exploration involves the exploration of the self and the employment environment, which focuses on carrying out career options, developing abilities, accumulating experiences, and reaching goals [26]. Students who actively explore their internal and external surroundings can consciously relate their motivations [44], interests, and abilities to acceptable occupational roles and engage in more goal-oriented behaviors than those who do not [45]. Through explorations of the self and the environment, students gain a full understanding of their internal characteristics and occupational traits [20], which helps them seize job opportunities and sustain their competitiveness in the labor market [46].

Drawing upon career construction theory, we examine the role of the future work self in inspiring career exploration and, subsequently, driving perceived employability among students. Career construction theory posits that career development is an action-oriented process in which individuals establish careers and design their own lives [47]. Individuals who are willing or flexible to make changes are more likely to engage in career-related activities [19]. Meanwhile, the career construction model of adaption divides the adaptive construct process into four links: adaptive readiness, adaptability resources, adapting responses and adaptation results [19]. Career exploration is an important expression of adaptive response, which can help students better cope with career development tasks and changes in the job market environment [11, 23]. Specifically, future work self helps students envision desirable futures and highlights discrepancies between current and ideal states. Recognizing these differences enables individuals to visualize the potential challenges they may encounter in pursuing their future career goals and to proactively explore opportunities in their career development process to prepare for these challenges [48]. Additionally, individuals actively use career resources to adapt to the demands of the dynamic work environment while constructing their careers [19]. Based on this framework, the future work self enables students to explore and rediscover themselves through the process of identity construction and to actively work toward a future that is consistent with their goals [48]. As it constitutes the positive possible selves and is a motivational resource in the context of work [11], studies have suggested that the future work self is positively linked to proactive career behaviors [16, 49], such as career planning and skill development. Moreover, when shaping their future work self, students are more likely to seek relevant information and suggestions on environmental clues [15], which not only provides a clearer image of their occupational self-concept [50] but also forms “personalized” career planning for constructing themselves [51]. Therefore, it can be inferred that there is a positive correlation between the future work self and career exploration.

In today’s complex, dynamically shifting labor market, it is crucial to hold a positive perception of employability. A proper assessment of employability can help students proactively choose the right career path that suits their career planning [52] and cope with work-related challenges and unexpected job transitions [53]. Scholars have shown that career preparatory behaviors (e.g., exploration) can lead to the development of career-related ideas and attitudes [54]. According to self-determination theory, when faced with an event that has a significant impact on their career, individuals are motivated to explore new ideas, adjust their behavior and engage with ongoing change to cope with the changing environment and achieve positive career-related outcomes [25]. When students experience a period of career role transformation and the transition from education to social work, they need to engage in more career exploration activities to actively seek career-relevant experiences [55], construct their possible selves, and clarify their career path [56]. Through career exploration, students re-examine themselves, strengthen their skills and formulate strategies to achieve goals [57], which in turn enhance their employability. This suggests that career exploration is positively associated with perceived employability.

From the standpoint of career construction theory, the concept of the future work self is seen as a valuable source of motivation that empowers individuals to invest greater effort in career-related actions and achieve favorable career results by continually developing and exploring future work objectives and strategies [11, 15, 16, 58]. Concurrently, according to the self-determination theory, individuals are inclined to actively explore and shape their present roles, leading to positive career outcomes, when they experience strong intrinsic motivation, such as that provided by the future work self [18, 26]. Thus, the future work self motivates students to consider their future aspirations, promotes meaningful career exploration behaviors, and thus enhances perceived employability. This means that the future work self is positively associated with career exploration, which, in turn, is positively related to perceived employability. Therefore, we propose the hypothesis as followed:

Hypothesis 2. Career exploration mediates the relationship between future work self and perceived employability.

### The moderating role of job market knowledge

From the perspective of career exploration, students exhibit variations in their career motivations. Research indicates that students’ engagement in career preparatory activities is influenced by their personal resources [59]. These resources can both trigger and constrain career preparatory behaviors [59], thereby impacting their career development and overall well-being [60]. As a personally relevant resource, job market knowledge plays an important role in judging the employment situation, making career decisions, and promoting career success [61]. Job market knowledge refers to the degree to which students are familiar with current labor market developments and future trends [62]. Research has demonstrated that students who acquire more job market knowledge in their education can be self-motivated to perform specific actions related to career development [63]. Thus, we posit that the positive impact of career exploration on the perception of employability can be reinforced when students possess a high level of job market knowledge.

According to career construct theory, the environment in which a career develops provides the driving force and guidance for how individuals construct their careers [19]. Being attentive to contextual cues allows individuals to actively process career-related information and make progress in their careers [20]. Accordingly, students who have well-equipped job market knowledge are more concerned about their future career direction, engage in more career exploration activities, and become more proactive in developing their careers than those who do not. This well-equipped understanding of the job market serves as a valuable resource, aiding students in better understanding themselves and the external environment [64], and as a result, enhancing their employability prospects. In contrast, when students possess less job market knowledge, they are blindly optimistic about the labor market and are more reluctant to break out of their comfort zone to carry out career strategies [65] and enhance their career competencies. As such, due to a lack of awareness of the job market, they exhibit fewer career exploration behaviors and are reserved in boosting their employability [66]. From the above analysis, we infer that the relationship between career exploration and perceived employability is enhanced when students have a higher level of job market knowledge. Accordingly, we propose the hypothesis as followed:

Hypothesis 3. Job market knowledge moderates the positive relationship between career exploration and perceived employability, such that when an undergraduate’s job market knowledge is higher, this relationship becomes stronger.

### The moderated mediation model

Based on the aforementioned hypothesis, we argue that job market knowledge moderates the indirect effects of the future work self on perceived employability through career exploration. Specifically, the influence of the future work self on perceived employability, via career exploration, is amplified when a student possesses a greater level of job market knowledge. According to career construction theory [19], students with sufficient job market knowledge have a clearer orientation of their future selves in the context of work [11]. Furthermore, they are more likely to engage in extensive career exploration in order to continuously comprehend their interests [67], motivations and career aspirations [57] compared to those who lack such knowledge. Consequently, their perceived employability is enhanced through the creation of a wider spectrum of future possibilities. Conversely, when students possess less job market knowledge, i.e., when they have less knowledge about the future labor market and the current employment situation, they have a vague self-image related to their future jobs [38] and engage in less career exploration behavior, thus limiting the development of their perceived employability. Drawing on these findings, we propose the hypothesis as followed:

*Hypothesi*s* 4*. Job market knowledge positively moderates the indirect relationship between future work self and perceived employability through career exploration, such that the relationship becomes stronger when an undergraduate’s job market knowledge is higher.

## Study 1

### Method

#### Sampling and procedure

We recruited a total of 65 students majoring in tourism management from a junior college located in central China to participate in a scenario experiment. After excluding four participants who failed the attention check question, we derived data from a valid sample of 61 individuals. Among these participants (*N* = 61), 21 were males (34.4%) and 40 were females (65.6%). Their average age was 19.31 years (*SD* = 1.36). Participants were randomly assigned to two scenarios (future work self: high vs. low). We manipulated different levels of future work self by means of scenario descriptions. After reading the experimental material on future work self, participants were asked to complete the future work self scale based on the scenario material read above. Immediately following this, participants were asked to report information on other variables (career exploration and employability) and provide demographic information based on their true feelings in the scenario. At the end of the experiment, participants were rewarded with a bonus pack.

### Manipulation and measures

We developed experimental materials for future work self based on the research by Strauss and Parker [68]. The specific content of the experimental material was in the appendix.

Future work self. After reading the experimental materials, participants were asked to complete the 5-item scale developed by Strauss et al. (2012) which was widely used to measure future work self in previous studies [11]. A representative item was “I am very clear about who and what I want to become in my future work *(1* = *strongly disagree, 7* = *strongly agree).*” The Cronbach’s α was 0.95.

Career exploration. We used the 12-item scale by Stumpf, Colarelli, and Hartman (1983) to access career exploration [69]. A 7-point scale was used *(1* = *strongly disagree to 7* = *strongly agree)* to show the extent to which participants agreed with each item (e.g., “I prepared mentally for my work”). The Cronbach’s α was 0.66.

Perceived employability. We used a 16-item scale developed by Rothwell, Herbert, and Rothwell (2008) to measure student’s employability [32]. A sample item was “The knowledge and skills I possess are what employers are looking for *(1* = *strongly disagree, 7* = *strongly agree).*” The Cronbach’s α was 0.85.

### Result

#### Manipulation check

The results of the ANOVA indicated that participant’s perceived level of future work self was significantly higher in the high level of future work self condition (*M* = 6.49, *SD* = 0.47) than in the low level of future work self condition (*M* = 2.50, *SD* = 0.45), and the difference between the two conditions was significant (F(1, 59) = 1143.33, *p* < 0.001, ${\eta }_{p}^{2}$= 0.95). Thus, we successfully manipulated the future work self.

### Hypotheses testing

Descriptive statistics such as mean, standard deviation and correlation coefficients of the variables were given in Table 1.

**Table 1:** Descriptive statistics analysis and correlation coefficients (study 1)

Variable	Mean	SD	1	2
1. Future work self ^a^	4.53	2.06	-	
2. Career exploration	3.17	0.62	0.33^**^	-
3. Perceived employability	3.27	0.73	0.43^**^	0.85^**^

*N* = 61. ^a^ 0 = low future work self condition (*n* = 30), 1 = high future work self condition (*n* = 31). ^**^p < 0.01

First, we conducted a one-way ANOVA with future work self as the independent variable and employability as the dependent variable. The results showed that different levels of future work self had significantly different effects on students’ perceived employability (F(1, 59) = 14.42, *p* < 0.001, ${\eta }_{p}^{2}$= 0.20). Specifically, the high level of future work self condition (*M* = 3.59, *SD* = 0.61) led to the higher level of perceived employability compared to the low level of future work self condition (*M* = 2.94, *SD* = 0.91). Therefore, hypothesis 1 was verified.

Second, we used PROCESS to conduct mediation effect test. The results showed that future work self was significantly and positively correlated with career exploration (*b* = 0.43, *p* < 0.01). Career exploration was significantly and positively associated with employability (*b* = 0.93, *p* < 0.001). Bootstrapping results from a sample of 5,000 showed that the indirect effect of future work self on perceived employability via career exploration was 0.40. And the bootstrapped confidence interval [95% CI: (0.12,0.68)] did not include zero. Thus, the mediating effect was significant, supporting hypothesis 2.

### Discussion of study 1

A scenario-based experimental approach was employed in Study 1 to test the model. The experimental results showed that the main and mediating effects of the theoretical hypotheses model proposed in this study were valid. The experimental study tested the causal relationships between the independent and mediating variables, and between the dependent and outcome variables, further enhancing the validity of the findings of the study on the mechanism of the influence of future work self on students’ perceived employability. To further test the impact of the moderating variables, Study 2, a questionnaire study, was conducted.

## Study 2

### Sampling and procedure

We used the time-lagged research design to collect data via submitting questionnaires among Chinese undergraduates at a university in the middle area of China. One of the authors, as a teaching assistant of a career development course at this university, extended an invitation to the undergraduate students to complete the questionnaires in the classroom. Specifically, the author introduced the topic of this study to 495 students who majored in hospitality and tourism, and asked them to participate in this study. After receiving conformation to participate from 288 undergraduates, the author submitted the online questionnaire to them. Specifically, the questionnaire was uploaded to Wenjuanxing which is an online questionnaire system widely used in academic study in China. The author subsequently shared the questionnaire link, generated by Wenjuanxing, with the students on WeChat, the most prominent Chinese social media platform, and invited them to participate in filling out the questionnaire on WeChat. The time-lagged research design was employed in the current study with an eight-week time interval. At time 1 (T1), these undergraduates were asked to report their future work self, career exploration, job market knowledge, and their demographic information. Eight weeks later, at time 2 (T2), they were asked to rate their perception about their employability. After matching their two sets of responses, a valid sample of 253 undergraduates were used in the study. Of those reporting, participants included 153 males (60.5%) and 100 females (39.5%), and their average age was 21.68 years old (*SD* = 3.20). The sample consisted of 70.8% undergraduate students (*N* = 179), 18.5% master students (*N* = 47) and 10.7% doctoral students (*N* = 27).

### Measures

All the measurement scales are mature English scales, and the translation-back translation method is used to ensure the Chinese versions can accurately express the original concepts [70]. Before the formal distribution of the questionnaires, we invited undergraduates to take a pre-survey and revised certain items that were inaccurately stated, inappropriate and difficult to understand based on undergraduates’ feedbacks and experts’ advice.

Future work self and career exploration scales used in Study 2 were all consistent with those used in Study 1. The Cronbach’s α for the two scales were 0.91 and 0.92. We employed the three-item scale developed by Hodzic, Ripoll, Lira, and Zenasni (2015) to measure undergraduate’s employability [71]. The scale combined with the Likert-7 point scoring method has been widely used to evaluate employability (*1 = strongly disagree to 7 = strongly agree*). A representative item was “in the current job market situation, I think it is possible to find an interesting job.” The Cronbach’s α of this scale was 0.90. Job market knowledge was measured using a three-item scale compiled by Hirschi, Nagy, Baumeler, Johnston, and Spurk (2018) [72]. Participants were asked to rate a 5-point Likert scale of 1 = strongly disagree to 5 = strongly agree. The representative item was “I have a good knowledge of the job market.” The Cronbach’s α for job market knowledge was 0.96.

We controlled respondents’ age, gender, and education level. According to previous studies, an individual’s employability increases with age [73] and the level of education [74]. We also noticed that women appear to be more confident in their employment opportunities when they are unemployed [75]. Therefore, we statistically controlled these variables for their potential influences. In addition, since researchers have suggested that such environmental-oriented factor as supports from family and schools may exert influences on students’ career-related behaviors and attitudes during school-to-work transition (e.g., perceived employability, and career explorations) [76], we in the current study controlled career support from school by using the 6-item scale from Sturges et al. (2002) [77]. A representative item was “I have been given training to help develop my career.” The Cronbach’s α of this scale was 0.77.

### Analytical strategy

Firstly, we conducted reliability and validity tests on the data using SPSS 26.0 and AMOS 26.0. Next, we use hierarchical regression to test for mediating and moderating effects with SPSS 26.0 to support the hypotheses. Finally, to further elucidate the indirect effect and the validation of the result, we used the PROCESS procedure by Hayes developed in SPSS [78] to generate a confidence interval (CI) using a bootstrap program with 5000 sample size.

## Results

### Confirmatory factor analysis

Before testing hypotheses, we adopted AMOS 26.0 to conduct confirmatory factor analysis on four variables: future work self, career exploration, job market knowledge, and perceived employability to examine the discriminant validity among the variables. As shown in Table 2, the four-factor model was significantly better than the other competing models and demonstrated a good fit (χ^2^/*df* = 2.79, *CFI* = 0.93, *RMSEA* = 0.08, *IFI* = 0.93, *TLI* = 0.91), which indicated that the variables of the measurement model have good discriminant validity.

**Table 2:** Confirmatory factor analyses (study 2)

Model	$\frac{{\upchi }^{2}}{df}$	RMSEA	CFI	IFI	TLI	Δχ2
Four-factor model	2.79	0.08	0.93	0.93	0.91	—
Three-factor model	6.81	0.15	0.73	0.73	0.70	989.94^***^
Two-factor model	8.94	0.18	0.63	0.63	0.59	502.14^***^
Single-factor model	10.27	0.19	0.56	0.57	0.52	314.96^***^

*N* = 253; ^***^p < 0.001

Four-factor model: Future work self, Job market knowledge, Career exploration, Perceived employability

Three-factor model: Future work self + Career exploration, Job market knowledge, Perceived employability

Two-factor model: Future work self + Job market knowledge + Career exploration, Perceived employability; Single-factor model: Future work self + Job market knowledge + Career exploration + Perceived employability

Collinearity evaluation is also carried out to find out whether there is collinearity in the model. To test collinearity, VIF calculation is needed for each construct. If the VIF score is higher than 5, then the model has collinearity in the educational psychology domain [79]. The results of the collinearity assessments showed that all VIF scores were less than 4.4, meaning that no pathological collinearity issue existed in the model.

In addition, since the data was collected form only one source (i.e., students), we conducted two methods to identify the potential for common method bias (CMB). We first followed the explanatory factor analysis from Harman (1976) [80], and the results showed that one factor accounted for 31.25%, which is below the accepted threshold of 40%. Meanwhile, we conducted the test of the one-factor measurement model [81], generating a poor fit to the data. Taken together, CMB is not a serious problem in our study.

### Descriptive statistics

Table 3 presents the results of descriptive statistics such as the mean, standard deviation, and correlation coefficient of the variables. In line with our expectations, the results of Pearson correlation analysis show that future work self is significantly related to career exploration (*r* = 0.61, *p* < 0.01) and is positively related to perceived employability (*r* = 0.48, *p* < 0.01). Moreover, career exploration presents a positive relationship with participants’ perceived employability (*r* = 0.51, *p* < 0.01). These results give initial support for the hypotheses.

**Table 3:** Descriptive statistics analysis and correlation coefficients (study 2)

Variable	Mean	SD	1	2	3	4	5	6
1. Future work self	4.13	1.33	-					
2. Career exploration	4.81	1.07	0.61^**^	-				
3. Job market knowledge	2.92	1.03	0.58^**^	0.61^**^	-			
4. Perceived employability	4.68	1.30	0.48^**^	0.51^**^	0.42^**^	-		
5. Gender	1.40	0.49	0.02	0.02	-0.07	-0.02	-	
6. Age	21.68	3.20	0.15^*^	-0.04	0.15^*^	0.02	0.02	-
7. Education	3.99	2.58	0.06	-0.08	0.05	-0.02	0.04	0.83^**^

*N* = 253; ^**^p < 0.01; ^*^p < 0.05

### Hypotheses testing

In the current paper, the hypotheses were verified by means of the hierarchical regression approach and Bootstrap method. We used SPSS 26.0 and the SPSS macro program PROCESS for data analysis. Table 4 reports our results and the specific analysis results are as follows.

**Table 4:** Hierarchical Regression Results (study 2)

Variable	Career exploration			Perceived employability				
	Model 1	Model 2		Model 3	Model 4	Model 5	Model 6	Model 7
Gender	0.05	0.02		-0.05	-0.08	-0.08	-0.05	-0.06
Age	0.02	-0.05		0.05	-0.01	0.01	0.02	0.02
Education	-0.05	-0.13		-0.06	-0.01	-0.01	-0.02	-0.02
Future work self		0.51^***^			0.48^***^	0.27^***^		
Career exploration						0.42^***^	0.51^***^	0.51^***^
Job market knowledge							0.20^*^	0.17
Career exploration × Job market knowledge								0.12^*^
R^2^	0.007	0.39		0.006	0.24	0.31	0.28	0.30
△R^2^	0.007	0.38		0.006	0.23	0.07	0.27	0.02
F	0.59	39.90^***^		0.47	19.22^***^	22.03^***^	19.13^***^	17.35^***^

*N* = 253; ^***^p < 0.001; ^*^p < 0.05

As shown in Model 4 in Table 4, when participants’ gender, age, and education were controlled for, the positive effect of future work self on undergraduate’s perceived employability is significant (*b* = 0.35, *p* < 0.001). Thus, the results support hypothesis 1.

After considering all the control variables, the results shown in Table 4 Model 2 indicate that future work self is significantly and positively associated with career exploration (*b* = 0.421, *p* < 0.001) As shown in Table 4 Model 5, career exploration positively affects undergraduates’ perceived employability after controlling for future work self (*b* = 0.40, *p* < 0.001). Further, we adopted the Bootstrap method to probe the indirect effects [82] and set 5000 bootstrapped samples. The results show that the indirect effect of future work self on undergraduate’s perceived employability via career exploration was 0.15 with a 95% confidence interval of [0.08,0.24], and the upper and lower intervals do not contain zero which suggests mediation is indicated. Therefore, hypothesis 2 is supported.

**Fig. 2:**
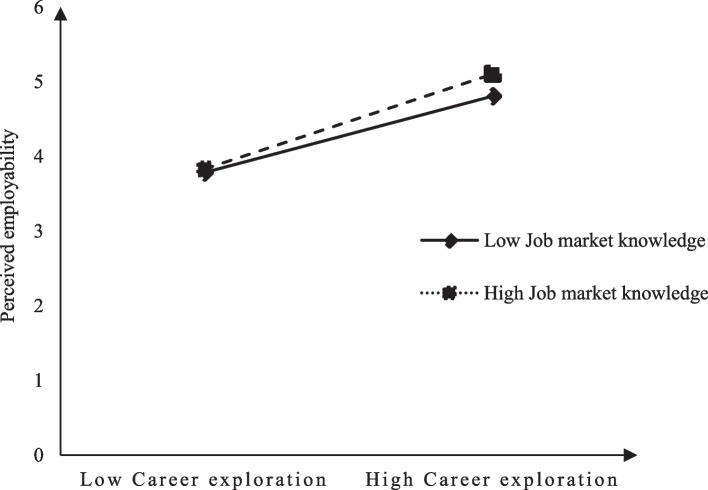
Interaction between career exploration and job market knowledge on perceived employability

Hypothesis 3 proposes that job market knowledge moderates the relationship between career exploration and perceived employability. As shown in Table 4 Model 7, the interaction term of “career exploration” × “job market knowledge” is significantly and positively related to the perceived employability (*b* = 0.14, *p* < 0.01). To further test the moderating effect of job market knowledge, interaction effects are plotted at high (+ 1 SD) and low (-1 SD) levels of job market knowledge. As showed in Fig. 2, a simple slope test reveals that career exploration shows a significant tendency to enhance perceived employability at high levels (*b* = 0.61, *t* = 6.40, *p* < 0.001) and low levels (*b* = 0.35,* t* = 3.88, *p* < 0.001) of job market knowledge. Thus, hypothesis 3 is supported.

Further, we employed the Bootstrap method to test for moderated mediation effect and set 5000 repeated sampling times to obtain an indirect effect and 95% confidence intervals for future work self on perceived employability when job market knowledge is one standard deviation higher or lower than the mean. As can be seen from Table 5, for undergraduates with less job market knowledge, the indirect effect is 0.13, and the bootstrapped confidence interval (95% CI: [0.01, 0.23]) excludes zero. For undergraduates with medium job market knowledge, the indirect effect was 0.19, with a 95% confidence interval [0.07,0.27], excluding zero. For undergraduates with high job market knowledge, the indirect effect is 0.26, and a 95% confidence interval is [0.12, 0.32] excluding zero. The indirect effect of the difference between two conditions (high and low conditions of job market knowledge) is 0.16 with a 95% confidence interval of [0.01, 0.30]. The interval excludes 0 and the difference is significant. In conclusion, job market knowledge significantly moderates the indirect effect of future work self on undergraduates’ perceived employability. Hypothesis 4 is verified.

**Table 5:** Analysis of the moderated mediating effect (study 2)

	Indirect Effect	Boot SE	95% CI
Low level of job market knowledge (-1 SD)	0.13	0.07	[0.01, 0.27]
Medium level of job market knowledge (Mean)	0.19	0.05	[0.09, 0.30]
high level of job market knowledge (+ 1 SD)	0.26	0.05	[0.16, 0.36]
difference between high and low	0.16	0.07	[0.01, 0.30]

*N* = 253; Bootstrap sample size = 5000

### Discussion of study 2

Study 2 used a questionnaire method to test the overall model and the data results supported the hypotheses of this study. The findings revealed that the positive relationship between future work self and employability was mediated by career exploration. In addition, job market knowledge positively moderated the indirect relationship between future work self and perceived employability through career exploration, such that the relationship became stronger when an undergraduate’s job market knowledge is higher.

## Discussions

### Overview of findings

Aiming at examining how and when undergraduates’ future work self contributes to their perceived employability by utilizing career construction theory and self-determination theory, the current research conducts two independent studies (i.e., an experimental study and a time-lagged field study) to investigate the role of career exploration as a mediator and job market knowledge as a moderator. The results indicate that undergraduates’ future work self is positively related to their perceived employability through increasing their career exploration. In addition, when undergraduates’ job market knowledge is high, their career exploration is more likely to boost their employability, and their future work self is also more likely to improve their employability via enhancing their career exploration.

### Theoretical implications

By employing career construction theory and self-determination theory, we provide theoretical implications. First, the results demonstrate that individuals’ positive self-concepts have significant and beneficial effects on the development of undergraduates’ perceived employability [83]. Specifically, the typical self-concept—i.e., the future work self—highlighting future orientations represents individuals’ strong motivations, perceptions, and behaviors [84], which effectively guides individuals’ processing of self-relevant information toward a better outcome [85]. In the domain of career development, since graduates’ future work selves serve as guides or references for developing their career-related abilities, knowledge and skills in the future workplace [28], undergraduates with a high level of future work self have identity-based motivation toward career planning, skill development, and networking [16]. That is, their current career-related behavior is consistent with their characteristics and aimed toward the attainment of their desired future, such as being employable [86]. These findings are aligned with career construction theory [19], suggesting that positive self-concepts tend to expand undergraduates’ aspirations and develop their thinking about future career possibilities [16]. It, thus, significantly allows them to redefine their future self and proactively promote their employability [38]. At the same time, these results are consistent with self-determination theory [22], which posits that positive intrinsic motivation (e.g., future work self) is an effective predictor of positive job and career outcomes (e.g., employability) [17].

Meanwhile, by integrating insights from career construction theory and self-determination theory, we propose and find a mediated relationship between the future work self, career exploration, and employability. That is, we contribute to unfolding the black box of behavioral processes by demonstrating that graduates’ future work self could trigger career explorative behaviors toward enhancing employability. Consistent with career construction theory and self-determination theory, which posit that career exploration is a key mediator in explaining the relationship between students’ career motivation and positive career outcomes [23–25], our findings indicate that individual self-factors with proactive motivations generate internal goals that boost career development behaviors [11, 16], which are conducive to positive career outcomes, such as employability [18, 46].

Our results also demonstrate that graduates’ job market knowledge positively moderates the relationship between the future work self and employability via career exploration. Specifically, an undergraduate who has a higher level of both future work self and job market knowledge is more likely to engage in career explorative behaviors, which in turn increases his or her employability in the future job market. These findings extend previous studies on treating career-related knowledge and skills as personally relevant resources by demonstrating that obtaining job market knowledge can strengthen individuals’ career behaviors and outcomes (e.g., judging the employment situation, making career decisions, and promoting career success) [61]. Existing research drawing on career construction theory has suggested that some environmental factors can be used to actively process career-related information and advance individuals’ career development [20]; that is, well-examined boundary conditions are context-oriented [87]. In the current study, we go one step further by showing that this motivational process (i.e., the future work self boosts employability by increasing career exploration) is further strengthened in the presence of boundary conditions such as individuals’ knowledge about their future jobs from the perspective of personal-dependent characteristics. Thus, we enrich the current understanding that undergraduates should have a comprehensive thought on their jobs and better understand what competencies, knowledge and skills are necessary to successfully search for a job to sustain their future employability.

### Practical implications

According to the findings in the current studies, there are some practical implications that can be provided. First, universities should emphasize on developing undergraduates’ future work self which is amenable to intervention and change [16, 88]. Specifically, counseling interventions and strategies can be designed for use with undergraduates facing career transitions, such as offering courses regarding to planning careers in the future and job searching strategies.

On the one hand, according to the mediator of career exploration, we encourage students to develop such proactive behaviors as exploring their future career. For example, after making a list of some possibilities of future jobs, students should engage more in activities of proposed career options (e.g., getting involved in the workplace for valuable insight into a career workday). On the other hand, it is suggested that educators and counselors guide students to identify the discrepancies between their current states and future resource requirements, which in turn stimulate students to take steps to cope with these challenges towards a more promising future. Meanwhile, educators and counselors could provide more external opportunities (e.g., building professional network) to evaluate students’ career interests, enhance students’ career abilities for their future their career choices. In this vein, students could discover the jobs that are available to them after their graduation from universities.

Finally, some job lessons should be integrated into current course designs [21]. For example, teachers in universities should be trained to employ active learning methods in class towards supporting students to develop a realistic perception of the job market in their community as well as to their own interests and strength. In this vein, undergraduates can be well prepared to make realistic and personal decisions regarding their educational and professional future.

### Limitations and future research

Some limitations can be noted in the current research. First, we collected data from one source (i.e., undergraduates). Although we have tested that CMV is not a potential problem in the study, we encouraged future research to invite others to rate undergraduates’ employability (e.g., teachers), which would increase the objectiveness of the results. A related limitation is about broadening the samples. Specifically, since an increasing number of Chinese undergraduates are pursuing a master’s degree, it is highly recommended to replicate our results among postgraduates. Thus, the generalization of the results reported in the current study awaits further empirical examination.

In addition, to further improve the overall robustness and rigor of the current research, it is highly recommended to rate students’ employability by other’s rating. We in this research, theoretically, aim to investigate students’ personal perceptions of their employability in the future job market; thus, we invited them to rate their perceived employability. Empirically, our examinations also precluded the possibility of CMB. However, others’ rating would provide more valid results on students’ employability. For example, following Roessler, Brolin, & Johnson (1990) [89], researchers in the future could invited employers to assess students’ employability in the real workplace, which may reveal the extent to which students are employable.

The final limitation regards to the research design. Specifically, although we conducted two independent studies in the current research by employing the experimental design (i.e., Study 1) and the time-lagged design (i.e., Study 2), it limits our ability to determine the direction of causality among the variables to the most extent. For instance, the findings may be influenced by opposite or bidirectional relationships due to the potential for undergraduates who have explored their career to enhance their development of future work self. This is because individuals are able to thoroughly examine their internal attributes, which facilitates the formation of a clear self-image in relation to work [39, 90]. As research indicated the reciprocal relationship between future work self and career exploration [48], whether Chinese students’ future work self and their perceived employability are reciprocally related over time. Thus, we suggest that scholars in the future should conduct a more rigorous research design (e.g., the time-lagged research design) to further validate our research findings in terms of reciprocal relationship.

### Supplementary Information

**Additional file 1.** High future work self condition, Low future work self condition

## Data Availability

The data resulting from this study is stored and protected according to the Data Management rules of the School of Business and Economics of the Vrije Universiteit Amsterdam, and so are not publicly available. Data are however available from the authors upon reasonable request and with permission of the School of Business and Economics of the Vrije Universiteit Amsterdam.
