# Supplementary material for: Linking undergraduates’ future work self and employability: a moderated mediation model
Source: BMC Psychol. 2024 Mar 18;12:160. doi: 10.1186/s40359-024-01530-1 (PMC10949635; doi:10.1186/s40359-024-01530-1)
Supplement: Supplementary file 1 — Additional file 1. High future work self condition, Low future work self condition [file 40359_2024_1530_MOESM1_ESM.docx]

**Experimental material**

1. **High future work self condition**

Alice is an undergraduate majored in hospitality management in a Chinese university. She is a very future-oriented student in the university as she regularly has significant hopes and aspirations in relation to her future work after graduation. Specifically, she imagines the future work self she hoped to become, and sets goals to move towards the future she envisions. For example, Alice realizes that she would definitely join in the hospitality and tourism-related industries as a tour guide in the future. It is easy for her to imagine that she, with a strong aspiration of showing visitors around places of interest, plans to be a historical guide by leading tourists around historical landmarks and points of interest like ruins, temples, battlefields and other sites of historical importance. She feels excited when she thinks about meeting new people, travelling, sharing incredible experiences and learn more about a culture or place in the future.

Meanwhile, as Alice sets a goal of being a historical tour guide after graduation, she compares her current situation with the requirements of being a successful tour guide in the future. Specifically, she must gain interesting facts and information about the area and events of the past that can appeal to sightseers; thus, she practices necessary skills required for this certain position. For example, she travels a lot in historical places towards gaining significant and factual expertise, practices her communication towards providing high quality customer service, and learn how to make scheduling for planning excursions in advance.

1. **Low future work self condition**

Alice is an undergraduate majored in hospitality management in a Chinese university. She is not a very future-oriented student in the university as the COVID-19 pandemic makes her feel hopeless about the future of joining in the hospitality and tourism-related industries. Thus, she has no significant hopes and aspirations in relation to her future work after graduation. Specifically, she never imagines the future work self she hoped to become, and doesn’t set goals to move towards the future she envisions. For example, although other classmates plan to be a tour guide after graduation, Alice realizes that she may not become a tour guide in the future; thus, she only focuses on studying in the classroom by leaning knowledge on hospitality and tourism management. She never thinks about whether and how the knowledge she obtained would benefit her career in the future, and she refuses to imagine her possible work in the future. Meanwhile, as Alice has not set a goal of a possible job after graduation, she has no sense of comparing her current situation with the requirements of a potential job position in the future.
